# Understanding Unmet Healthcare Needs in Nigeria: Implications for Universal Health Coverage

**DOI:** 10.1177/11786329251330032

**Published:** 2025-03-31

**Authors:** Paul Eze, Chioma Lynda Aniebo, Stanley Ilechukwu, Lucky Osaheni Lawani

**Affiliations:** 1 Department of Health Policy and Administration, Penn State University, University Park, PA, USA; 2 Department of Community Medicine, University of Nigeria Teaching Hospital, Ituku-Ozalla, Enugu, Nigeria; 3 Health Projects, South Saharan Social Development Organization (SSDO), Independence Layout, Enugu, Nigeria; 4 Institute of Health Policy, Management and Evaluation, University of Toronto, ON, Canada

**Keywords:** healthcare access, healthcare inequality, unmet healthcare needs, universal health coverage, Nigeria

## Abstract

**Background:**

Many individuals in low- and middle-income countries with healthcare needs do not access the necessary, often lifesaving healthcare services. Existing universal health coverage (UHC) indicators do not account for a portion of the population with unmet healthcare needs.

**Objective:**

To estimate the prevalence, wealth-related inequality, and determinants of unmet healthcare needs in Nigeria using data from the nationally-representative Nigeria Living Standards Survey, 2018-2019.

**Methods:**

We analyzed data from a cross-sectional sample of 116 320 Nigerians from 22 110 households selected using multi-stage probability sampling. The outcome variable was self-reported unmet healthcare needs. We conducted concentration index (CIX) analyzes to assess wealth-related inequalities and performed multilevel logistic regression analysis to identify the determinants of unmet healthcare needs at the individual, household, and community levels.

**Results:**

The prevalence of unmet healthcare needs was 5.2% (95% CI: 5.0-5.5), representing about 11 million Nigerians (95% CI: 10.5-11.5 million). The most common reasons were high costs (unaffordability) and the perception that the illness or injury was not serious. Wagstaff-normalized CIX for unmet healthcare needs was pro-poor: −0.09730 for the general population and −0.10878 for those with chronic illnesses. Significant determinants of unmet healthcare needs include age (AOR: 0.99, 95% CI: 0.99-1.00), chronic illness (AOR: 8.73, 95% CI: 7.99-9.55), single-person households (AOR: 1.55, 95% CI: 1.20-2.02), poorest quintile households (AOR: 1.45, 95% CI: 1.19-1.78), and mildly (AOR: 1.17, 95% CI: 1.01-1.36) or moderately food-insecure households (AOR: 1.30, 95% CI: 1.11-1.51).

**Conclusion:**

A significant proportion of Nigerians, particularly the very poor, chronically ill, those living alone, or food insecure, have unmet healthcare needs. This highlights the necessity for targeted interventions to ensure vulnerable populations can access essential healthcare services. To progress toward UHC, the Nigerian health system must address critical issues related to healthcare accessibility.

## Introduction

Achieving universal health coverage (UHC) – when all individuals receive quality healthcare without experiencing undue financial hardship – could transform health systems, especially for the poorest people, improve human capital, and spur economic growth.^1,2^ UHC enables people to receive timely and high-quality care, resulting in improved health outcomes and higher quality of life.^3^ UHC fosters social inclusion by guaranteeing that everyone, regardless of socioeconomic status, has access to essential health services.^2,4^ UHC plays a central role in achieving the Sustainable Development Goals (SDG).^5,6^ UHC incorporates 2 interconnected components: service coverage, which refers to the extent to which people receive the essential healthcare they need regardless of their ability to pay; and financial protection, which refers to the extent to which people are shielded from financial hardship while receiving healthcare.^7^ The UHC requires a delicate balance between the 2 components. When services are available but unaffordable, people cannot access care without suffering financial hardships. Conversely, if services are affordable but unavailable, individuals miss out on potential health benefits.^8^

UHC service coverage is measured as a geometric mean of 14 tracer indicators related to essential health services, such as immunization, maternal care, disease treatment, and healthcare infrastructure, while financial protection is measured using 2 key indicators: catastrophic health expenditure and impoverishing health expenditure.^7-11^ However, both measures of UHC only include people who have actually received healthcare services: service coverage measures the extent of healthcare services provided, while financial protection measures the extent of out-of-pocket (OOP) health payments that adversely impact households. These indicators do not account for individuals with healthcare needs who do not access services for various reasons, including their inability to afford OOP payments, physical distance, or unavailability of the services.^4,5,12-14^ For instance, a country might have a high service coverage index, yet still struggle with poorly addressed conditions, such as mental health or neglected tropical diseases – issues not captured by the 14 service coverage indicators.^13^ Additionally, a seemingly high financial protection index could mask low service utilization if people cannot afford the necessary services.^15^ Consequently, estimates obtained using these indicators underestimate the vulnerability of people who need healthcare services but cannot access them because of unavailability, unaffordability, distance, or even poor quality.^5,12-14^

The term “unmet need for healthcare” refers to various healthcare needs that are not adequately addressed or fulfilled.^13,16^ Unmet healthcare needs are an indicator of access to healthcare, as access is commonly understood as the fulfilment or realization of healthcare needs.^13,14,16,17^ The presence of unmet healthcare needs serves as a critical indicator for identifying barriers to accessing healthcare and for assessing existing inequalities within the healthcare system.^13^ Measuring healthcare needs, however, is challenging because they are embedded within social norms, the way we understand illness, and our perceptions of health.^13^ This is further complicated by several supply- and demand-side factors that hinder access to healthcare services, including geographic accessibility (physical distance or travel time to health facilities), service availability (clinic hours and waiting times), cultural and social acceptability, quality of care, and financial accessibility (affordability).^17^ In the sub-Saharan African context, however, it is the affordability of healthcare that is most pernicious. While cultural and educational factors, for example, might obscure the need for healthcare, unaffordability prevents utilization, even when the benefit of healthcare intervention is recognized.^18^ People with unmet healthcare needs are often aware that they are sick and that their condition may be treated, but they cannot access the necessary healthcare.^16^ Unmet healthcare needs represent a hidden population: individuals who silently suffer without seeking care. Over time, their health condition may deteriorate, resulting in significant health and economic consequences.^13,14,16,19^ Previous studies overwhelmingly indicate that unmet healthcare needs, in the low- and middle-income countries (LMIC), disproportionately affect low-income populations.^12,20-22^

Nigeria, Africa’s most populous country with over 220 million people, has made slow progress toward UHC.^23,24^ Nigerians face a significant risk of financial hardship due to healthcare expenses, as less than 3% of the population have any form of health insurance.^25^ Nigeria relies heavily on OOP payments for healthcare financing, with public financing covering only 25% of the total health spending.^23,26^ This reliance on OOP makes price a critical factor influencing healthcare demand, particularly in vulnerable Nigerians.^18^ However, approximately 30.8% of Nigerians live in extreme poverty, surviving on less than $2.15 per day.^24^ Administratively, Nigeria is divided into 36 states, grouped into 6 geopolitical regions: North-West, North-Central, North-East, South-West, South-South, and South-East. The South-West is the most developed, while the North-East is the least developed.^27^ In this study, we leveraged secondary data from the nationally representative Nigeria Living Standards Survey (2018-2019) to estimate the prevalence of unmet healthcare needs in Nigeria. Additionally, we examined wealth-related inequalities and determinants of unmet healthcare needs at individual, household, and community levels. Our study uniquely complements ongoing efforts to monitor progress toward UHC by providing a more nuanced and complete understanding of the gaps in healthcare access, ultimately supporting more effective strategies to achieve UHC. Our findings could provide valuable insights for guiding policy interventions aimed at ensuring equitable access to healthcare in Nigeria.

## Methods

### Study Design

**Figure 1.**
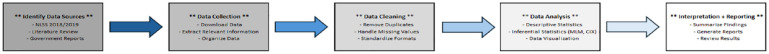
Flow-chart showing the data collection and analysis pipeline.

This was a population-based cross-sectional survey of Nigerians conducted between September 2018 and September 2019. The study considered all Nigerians eligible for inclusion, as every individual could potentially have unmet healthcare needs. We followed the Strengthening the Reporting of Observational Studies in Epidemiology (STROBE) guidelines to ensure appropriate reporting of the study’s design, conduct, and findings.^28^ Figure 1 summarizes our data collection and analysis pipeline.

### Data Source and Sample Size

The Nigeria Living Standards Survey 2018-2019 (NLSS) is a nationally representative survey of the living conditions of civilian households and communities in Nigeria (all 36 states and the Federal Capital Territory (FCT), Abuja), designed by Nigeria’s National Bureau of Statistics in collaboration with the World Bank.^29^ Initially designed to sample 600 households in each state (22 200 households nationwide), the insecurity challenges in Borno State limited the final sample to 22 110 households. The NLSS 2018-2019 used the enumeration areas (EAs) specified by Nigeria’s National Population Commission as the sampling frame. A 2-stage sample design was employed: In the first stage, 60 EAs were selected in each state (and FCT Abuja) using random systematic sampling. In the second stage, 10 households were selected in each EA using systematic sampling. Data were collected through face-to-face interviews conducted over a 12-months period (September 2018-September 2019). The information was recorded using highly automated computer-assisted personal interviewing devices, which allowed for real-time quality checks. The 2018-2019 NLSS was subsequently cleaned and comprehensively reviewed by data editors and assistants for inconsistencies and extreme values, and to ensure quality control.^29^ Since our study utilized secondary data, we did not conduct a traditional power analysis to determine the sample size. Secondary data, which comprises pre-existing data collected for other purposes, typically comes with fixed sample sizes that cannot be altered. Nevertheless, our study sample of 116 320 individuals across 22 110 households is sufficiently powered to achieve a 99% confidence level with a 1% margin of error.

### Dependent Variable

The outcome variable was constructed from the responses to 2 sets of questions. (1) *Has [NAME] consulted health practitioner or dentist or traditional healer or Patent Medicine Vendor or visited a health cent*e*r in the last 30* *days?* (2) *Was [NAME] sick or injured in the last 30* *days?* There were 2 possible response options for both questions: “Yes” or “No.” Those who responded “No” to the first question but “Yes” to the second question were classified as having unmet healthcare needs. Therefore, individuals are defined as having an unmet healthcare need if they fulfill 2 criteria: (1) reported a health shock and (2) could not seek healthcare. A similar set of questions has been previously validated as reliable for estimating the unmet need for healthcare.^22,30,31^

All respondents who indicated that they had an unmet need for healthcare were asked to provide further context. *Why did [NAME] not consult anyone regarding the most severe illness/injury specified in [Q6]?* There were 5 different response options for this question: (1) “No need, minor illness, or injury,” (2) “too far,” (3) “too expensive,” (4) “poor quality of care,” and (5) “Other.” The responses were grouped into two broad categories: cost-related unmet need for healthcare that’s more directly connected with budget constraints, that is, the “too expensive” issue; and non-monetary unmet healthcare need due to other reasons not directly associated with budget constraint, that is, all the other 4 issues.

### Independent Variables

Based on Andersen’s healthcare access framework, which provides a conceptual framework for understanding the factors influencing healthcare service utilization,^32^ we incorporated variables that could predict the use of healthcare services. This approach allowed us to explore and analyze the complex interplay of predisposing factors, enabling factors, and perceived or actual needs in determining healthcare utilization. Hence, we included the following predisposing factors: age, sex, marital status, religion, education, employment status, residential area, and region; enabling factors: household size, sex of household head, household wealth quintile, food insecurity and health insurance coverage; and need factor: chronic illnesses. Food insecurity was assessed using the Household Food Insecurity Access Scale (HFIAS).^33^

### Data Analysis

We conducted a descriptive statistical analysis to present the sociodemographic characteristics of the survey participants and their unmet healthcare needs. Specifically, the Student’s *T*-test and Pearson’s chi-squared test were used as appropriate to compare the differences between the unmet and met healthcare need groups. We computed the asset index value for each household using a Principal Component Analysis (PCA). The assets included in estimating the index value were furniture, plastic chairs, mattress, bed, mat, sewing machine, gas cooker, stove, fridge, freezer, air conditioner, washing machine, bicycle, motorbike, cars, and other vehicles, generators, fans, radio, cassette recorders, Hi-Fi sound systems, microwaves, iron, TV sets, computers, DVD players, satellite dishes, smartphones, and regular mobile phones. Based on these asset index values, we categorized households into wealth quintiles: the first quintile represents the poorest 20% and the fifth quintile represents the wealthiest 20% of the population.

We examined the extent of wealth-related inequality in unmet healthcare needs using the concentration index (CIX), a widely used method for assessing the degree of equality in healthcare utilization based on economic status.^34^ We estimated the CIX for unmet healthcare needs in the general population and in those with chronic illnesses using the “conindex” syntax in Stata.^35^ The CIX accounts for the respondent’s socioeconomic status (in this case, household wealth) and is sensitive to changes in the distribution of households across socioeconomic groups. The CIX is visually represented on the *x*-axis as a ranking of respondents by their wealth, from the poorest to the richest, and on the *y*-axis as the cumulative percentage of unmet healthcare needs. The shape of the CIX curve indicates whether wealthier or poorer individuals disproportionately have an unmet need for healthcare. The CIX ranges from −1 to +1. A CIX of 0 indicated that unmet healthcare needs were evenly distributed across various socioeconomic groups. A positive CIX suggests that wealthier individuals experience more unmet healthcare needs, whereas a negative CIX indicates that unmet healthcare needs are more frequently experienced by poorer individuals. The Wagstaff-normalized CIX, which adjusts the standard CIX for binary outcome variables like ours, provides a more accurate and suitable measure for comparing health inequalities in unmet healthcare needs.^36^

We conducted multilevel logistic regression using the “melogit” command in Stata to identify the factors associated with unmet healthcare needs, as this outcome of interest was binary. We fitted 5 models. In the null model (Model 0) – the model analyzed without any predictor variables – we examined the random effect of between-cluster variability at the household, community, and state levels. The intraclass correlation coefficient (ICC) was estimated to determine whether a multilevel analytical approach was required.^37^ Null models with non-zero log-likelihood ratio and ICC > 0.05 hinted at the presence of significant within-level correlation. In Model 1, we included individual-level factors such as age (as a continuous variable), sex, marital status, religion, education, employment status, and chronic illnesses as categorical variables. In Model 2, we included household-level factors: household size, gender of the household head, wealth quintile, household food security, and health insurance. In Model 3, we included community-level factors such as residential area and region. In Model 4, the full or saturated model, we concurrently fitted the important characteristics of Models 0, 1, 2, and 3 to observe their net fixed and random effects. We computed the proportional change in variance and median odds ratio to understand the relationships between our variables, and to test the robustness of our model.^37^ We used the deviance statistics to determine whether the inclusion of new variables significantly improved model fit. We used the Akaike Information Criterion (AIC) and Bayesian Information Criterion (BIC) to identify the best model, selecting the 1 with the lowest AIC and BIC values. We weighed our analyses using NLSS 2018-2019 sample weights to account for the stratified clustered sampling design. We conducted all statistical analyses using Stata MP 18.0, considering an α (alpha) ⩽.05 as the significance threshold.

## Results

### Characteristics of Study Sample

A total of 116 320 individuals participated in this study, representing an estimated population of 210 210 114 after applying sampling weights. The weighted mean age of study participants was 23.7 (95% CI: 23.6–23.9) years. Approximately half (50.3%) of the respondents were children (under 18 years old), and only approximately 7% were aged 60 years or older – Table 1. Females slightly outnumbered males (50.6% vs 49.4%, respectively). About one-third (32.2%) of the study participants were married; approximately 44.0% employed; and approximately 14.0% had 1 or more chronic illnesses. Most study participants (88.9%) lived in male-headed households, and few (2.4%) live in single-person households. Only 2.8% of the study participants had health insurance coverage, and almost three-quarters (73.9%) lived in food insecure households. Most study participants (72.2%) resided in rural areas, while 27.8% lived in urban areas.

**Table 1.** Sociodemographic characteristics of study participants (N = 116 320).

Sociodemographic characteristics	Frequency	Percentage
Sex		
Female	58 840	50.6
Male	57 480	49.4
Age (y)		
<5	16 657	14.3
5-17	41 825	36.0
18-39	33 738	29.0
40-59	16 101	13.8
⩾ 60	7999	6.9
Marital status		
Single/Never married	72 982	62.7
Married/Living as married	37 456	32.2
Widowed/Divorced/Separated	5882	5.1
Religion		
Islam	60 702	52.2
Christian	54 741	47.1
Others (African trad religion, none, etc)	857	0.7
Education		
None	69 111	59.4
Primary	23 705	20.4
Secondary	16 212	13.9
Post-secondary	7292	6.3
Employment		
No	65 091	56.0
Yes	51 229	44.0
Chronic illness		
No	100 032	86.0
Yes	16 288	14.0
Household head		
Female	12 960	11.1
Male	103 360	88.9
Median (IQR) household size	7 Persons	IQR: 5-9
Health insurance		
No	113 096	97.2
Yes	3224	2.8
Household food security		
Food secure	30 340	26.1
Mildly food insecure	27 967	24.0
Moderately food insecure	30 521	26.3
Severely food insecure	27 492	23.6
Residence		
Rural	83 943	72.2
Urban	32 377	27.8
Geopolitical region		
North-Central	23 682	20.4
North-East	23 008	19.8
North-West	28 088	24.2
South-East	13 135	11.3
South-South	14 978	12.9
South-West	13 429	11.5

### Prevalence and Reasons for Unmet Healthcare Needs

**Figure 2.**
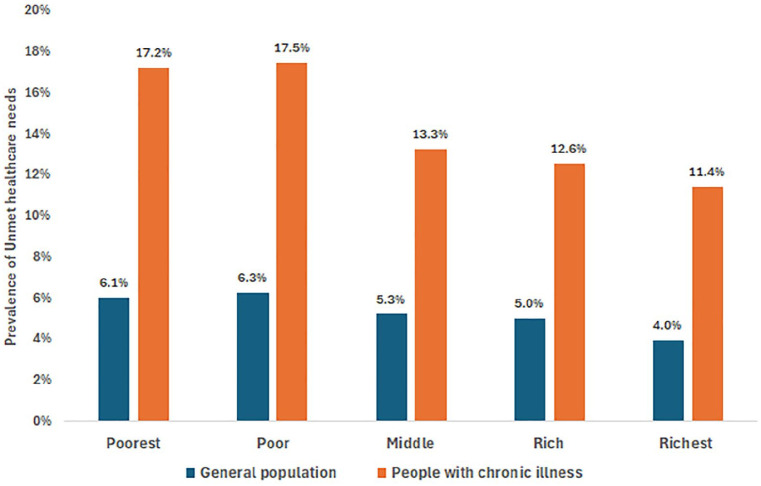
Prevalence of unmet healthcare needs by wealth quintile in the general population versus people with chronic illnesses.

**Figure 3.**
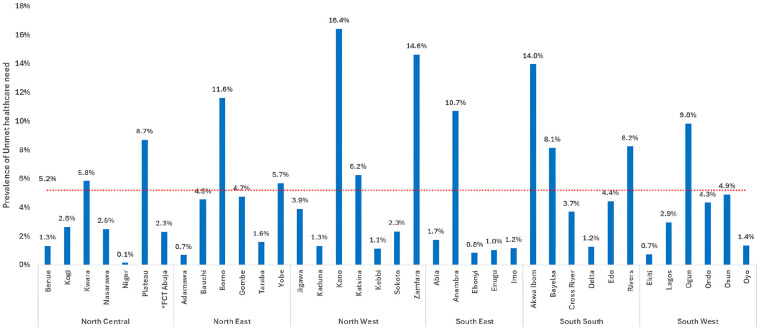
Prevalence of unmet healthcare needs in the 36 states and FCT Abuja, grouped by geopolitical region.

**Figure 4.**
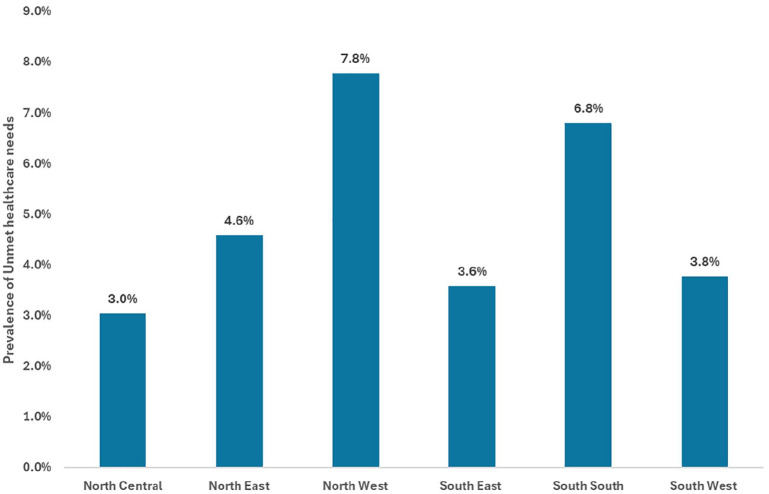
Prevalence of unmet healthcare needs in the 6 geopolitical regions.

The prevalence of unmet healthcare needs in this study was 5.2% (95% CI: 5.0-5.5), representing 11 million Nigerians (95% CI: 10.5 -11.5 million). However, the prevalence was nearly 3 times higher among individuals with chronic illnesses at 14.0% (95% CI: 13.3-14.8). Figure 2 shows that unmet healthcare needs were disproportionately prevalent among the poor (6.3%) and poorest (6.1%) quintiles compared with the richest (4.0%). A similar distribution across wealth quintiles was observed among study participants with chronic illnesses: the prevalence of unmet healthcare needs among those with chronic illness is significantly higher among the poorer segments of the population compared to the wealthier segments. Kano State and Zamfara State in the North West region, 16.4% and 14.6%, respectively, had the highest prevalence of unmet healthcare needs (16.4%), while Niger State in the North Central region (0.1%), Ekiti State in the South West region (0.7%), and Adamawa State in the North East region (0.7%) had the lowest (0.1%) – Figure 3. At the regional level, the North West region had the highest prevalence of unmet healthcare needs at 7.8% (95% CI: 7.4-8.2), while the North Central region had the lowest at 3.0% (95% CI: 2.8-3.3). Notably, the South South region’s prevalence of unmet healthcare needs was higher than the national estimate, at 6.8% (95% CI: 6.4-7.3) – Figure 4. The prevalence of unmet healthcare needs was higher among children under 5 years of age and among adults aged 40 years and older (*P* < .001) – Table 2. It was also significantly higher among people with chronic illnesses (*P* < .001), those living in single-person households (*P* = .013), and those living in food insecure households (*P* = .013) – Table 2.

**Table 2.** Association between predictor variables and unmet healthcare needs (incorporating sample weights).

	Unmet health care need		*P*-value**
Variables	No (n = 110 573) (%)	Yes (n = 5747) (%)	
Sex			
Female	55 862 (50.5)	2978 (51.8)	.303
Male	54 711 (49.5)	2769 (48.2)	
Age			
< 5 years	15 691 (14.2)	999 (16.8)	<.001
5-17 y	39 864 (36.1)	1961 (34.1)	
18-39 y	32 336 (29.2)	1402 (24.4)	
40-59 y	15 244 (13.8)	857 (14.9)	
⩾ 60 years	7438 (6.7)	561 (9.8)	
Marital status			
Single/Never married	69 549 (62.9)	3433 (59.7)	<.001
Married/Living as married	35 592 (32.2)	1864 (32.4)	
Widowed/Divorced/Separated	5432 (4.9)	450 (7.8)	
Religion			
Islam	57 343 (51.9)	3359 (58.4)	<.001
Christian	52 412 (47.4)	2349 (40.9)	
Others (African trad religion, none, etc)	818 (0.7)	39 (0.7)	
Education			
None	65 431 (59.2)	3680 (64.0)	<.001
Primary	22 620 (20.5)	1085 (18.9)	
Secondary	15 520 (14.0)	692 (12.0)	
Post-secondary	7002 (6.3)	290 (5.1)	
Employment			
No	61 878 (56.0)	3213 (55.9)	.379
Yes	48 695 (44.0)	2534 (44.1)	
Chronic illness			
No	96 512 (87.3)	3520 (61.2)	<.001
Yes	14 061 (12.7)	2227 (38.8)	
Household head			
Female	12 242 (11.1)	718 (12.5)	.290
Male	98 331 (88.9)	5029 (87.5)	
Household size			
1 person	2107 (1.9)	173 (3.0)	.013
2-5 persons	37 663 (34.1)	2002 (34.8)	
o > 5 persons	70 803 (64.0)	3572 (62.2)	
Wealth quintile			
Poorest	20 384 (19.9)	1251 (23.4)	<.001
Poor	20 464 (19.9)	1118 (20.9)	
Middle	20 543 (20.0)	1052 (19.7)	
Rich	20 556 (20.0)	1046 (19.6)	
Richest	20 719 (20.2)	884 (16.5)	
Health insurance			
No	107 517 (97.2)	5579 (97.1)	.638
Yes	3056 (2.8)	168 (2.9)	
Household food security			
Food secure	28 993 (26.2)	1347 (23.4)	.013
Mildly food insecure	26 476 (23.9)	1491 (25.9)	
Moderately food insecure	28 932 (26.2)	1589 (27.7)	
Severely food insecure	26 172 (23.7)	1320 (23.0)	
Residence			
Rural	79 759 (72.1)	4184 (72.8)	.053
Urban	30 814 (27.9)	1563 (27.2)	
Geopolitical region			
North-Central	22 950 (20.7)	732 (12.7)	<.001
North-East	21 856 (19.8)	1152 (20.1)	
North-West	26 210 (23.7)	1878 (32.7)	
South-East	12 719 (11.5)	416 (7.2)	
South-South	13 964 (12.6)	1014 (17.6)	
South-West	12 874 (11.6)	555 (9.7)	

** Incorporating sample weights.

One in 6 (16.4%) participants, representing 1.8 million Nigerians, had unmet healthcare needs due to financial constraints, “too expensive” – Table 3. Study participants who cited this reason were mostly in the poorest quintile – Appendix 1. Among those with chronic illnesses, the proportion of people with unmet healthcare needs due to financial constraints was even higher (21.7%) – Table 3. However, most participants (81.4%) stated that their illness was not severe enough to warrant medical attention. Additionally, many participants (4.6%), mostly in rural areas (*P* < .001), faced unmet healthcare needs due to the excessive distance to healthcare services, “too far” – Appendix 1. Appendix 2 and 3 presents the breakdown of the reasons for unmet healthcare needs by regions and states, respectively.

**Table 3.** Reasons for unmet healthcare needs in the general population versus in people with chronic illness (incorporating sample weights).

	General population		People with chronic illness	
Reasons	Proportion (%)	95% CI	Proportion (%)	95% CI
Cost-related unmet need for healthcare	16.4	14.5-18.5	21.7	18.8-24.9
Non-monetary unmet healthcare need				
1. Illness or injury not too severe	81.4	79.2-83.4	76.0	72.7-78.9
2. Health facility too far	4.6	3.8-5.5	5.6	4.3-7.2
3. Perceived poor quality	1.1	0.7-1.8	1.5	0.9-2.6
4. Other reasons	4.3	3.4-5.1	7.4	5.3-8.7

### Wealth-Related Inequality in Unmet Healthcare Needs

**Figure 5.**
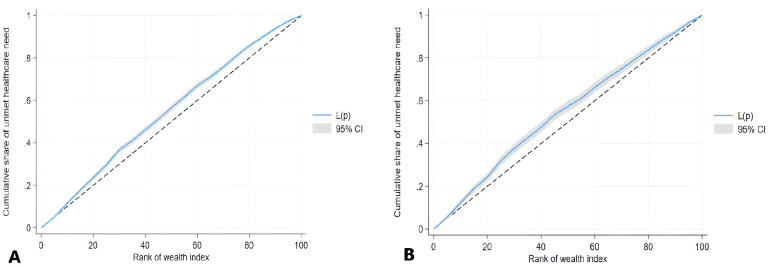
Concentration curve of unmet healthcare needs in the general population (A) versus people with chronic illnesses (B).

The standard CIX values for unmet healthcare needs are negative, −0.09223 for the general population, and −0.09356 for those with chronic illnesses, indicating that unmet healthcare needs are concentrated among less wealthy households – Table 4. The Wagstaff-normalized CIX, which adjusts the standard CIX for binary outcome variables such as ours, was higher: −0.09730 for the general population, and −0.10878 for those with chronic illnesses. Figure 5 shows that the curve plots for the cumulative percentage of unmet healthcare needs in both the general population (Figure 5A) and among those with chronic illnesses (Figure 5B) were above the line of absolute equality (the 45° line). This demonstrates that unmet healthcare needs is “pro-poor,” meaning that the incidence of unmet healthcare needs is more concentrated among the most economically disadvantaged segment of the population.

**Table 4.** Concentration indices of unmet healthcare needs in the general population versus in people with chronic illness.

	General population			People with chronic illness		
Concentration Index	Index value	Robust std. error	*P*-value	Index value	Robust std. error	*P*-value
Standard concentration index	−0.09223	0.013092	<.001	−0.09356	0.016236	<.001
Generalized concentration index	−0.00478	0.000679	<.001	−0.01309	0.002272	<.001
Erreygers normalized concentration index	−0.01913	0.002715	<.001	−0.05236	0.009087	<.001
Wagstaff normalized concentration index	−0.09730	0.013808	<.001	−0.10878	0.018877	<.001

### Factors Associated With Unmet Healthcare Needs

Our full model (Model 4) was considered the best-fit model as it had the smallest deviance statistic, AIC, and BIC – Appendix 4. As shown in the full model in Appendix 5, age, chronic illnesses, household size, wealth quintile, and food insecurity were significantly associated with unmet healthcare needs. For each 1-year increase in age, the odds of having unmet healthcare needs decreased by 1% (AOR: 0.99, 95% CI: 0.99-1.00). Adjusted results showed that having chronic illnesses (AOR: 8.73, 95% CI: 7.99-9.55), living in a single-person household (AOR: 1.55, 95% CI: 1.20-2.02), living in the poorest quintile household (AOR: 1.45, 95% CI: 1.19-1.78), and living in mildly (AOR: 1.17, 95% CI: 1.01-1.36) or moderately food-insecure households (AOR: 1.30, 95% CI: 1.11-1.51) were associated with unmet healthcare needs. Although differences in unmet healthcare needs based on employment status, gender of the household head, food insecurity, and household wealth quintiles were observed in Models 1 and 2 (Appendix 5) and differences based on religion, marital status, education, and region were observed in bivariate analyses (Table 2), these factors were not retained after adjustment in the full (saturated) model.

## Discussion

In this study, we estimated the prevalence of and wealth-related inequalities in unmet healthcare needs in the general population and among those with chronic illnesses. We also identified the reasons for unmet healthcare needs and examined the determinants at individual, household, and community levels. Our results reveal that a significant portion of Nigerians with healthcare needs cannot access the necessary and often lifesaving healthcare. Approximately 11 million Nigerians have unmet healthcare needs for various reasons. This finding complicates recent reports indicating that 23.8% of households in Nigeria experienced catastrophic healthcare expenditures, and that OOP health payments increased the poverty headcount by 0.8% pushing approximately 1.3 million Nigerians into poverty.^38,39^ Our findings also indicate that poor Nigerians disproportionately experience unmet healthcare needs. This finding was corroborated by our analysis of the underlying reasons for unmet healthcare needs, which showed that the poorest individuals cited financial constraints most frequently as the reason for their unmet healthcare needs. This study further indicates that enabling factors, such as socioeconomic status, household size, and household food security, along with need factors, such as having chronic illnesses, are the main contributors to unmet healthcare needs.

The prevalence of unmet healthcare needs in Nigeria is 5.2%, which is higher than 3.7% in Ghana,^40^ 2.5% in Thailand,^31^ 3.8% in Brazil,^21^ and 3.9% in Ireland,^41^ but lower than 8.0% in Liberia,^12^ 8.3% in Kenya,^22^ 10.5% in India,^42^ and 23.6% in Myanmar.^43^ Paradoxically, it was also lower than the prevalence in high-income countries with near-universal coverage: 7.6% in Japan,^44^ 7.8% in Canada,^45^ and 9.3% in South Korea.^46^ However, it is important to acknowledge the significant underreporting bias that may affect our prevalence estimate. The relatively high prevalence of unmet healthcare needs in Nigeria is attributed to several factors. The extremely low health insurance coverage plays a significant role. Unlike Brazil, Ghana, and Thailand, which have near-universal coverage, Nigeria’s lack of universal coverage and reliance on OOP payments deters vulnerable individuals from accessing healthcare services, even when they recognize the need for medical attention. In high-income countries such as Canada, Japan, and South Korea, countries with universal coverage, high unmet healthcare needs are attributed to aging populations and the increased demand for healthcare services among older adults who often have 1 or more chronic conditions.^45^ In Nigeria, extreme poverty, low education levels, poor understanding of ideal health, and limited availability of preventive, therapeutic, and rehabilitative healthcare services contribute to the high prevalence of unmet healthcare needs. Additionally, poor transportation infrastructure, especially in rural areas, further hinders Nigerians’ access to healthcare and worsens population health outcomes.^47^

The high prevalence of unmet healthcare needs among poor households, as observed in our analysis, aligns with the findings of other studies.^12,21,22,40,43,45,48,49^ Other studies using CIX analysis have also reported pro-poor inequality in unmet healthcare needs.^21,50^ This may be due to the fact that, without insurance coverage, most patients in Nigeria must pay out-of-pocket for outpatient care, medications, inpatient care, and even food during hospital stays.^26,51^ The combination of high OOP healthcare payments and limited financial resources makes it challenging for very poor households to access necessary healthcare services, leading to increased unmet healthcare needs.^39^ These households often have to choose between spending their limited funds on healthcare or other essential needs, such as food and housing.^18^ This financial strain often leads households to downplay such needs as “illness or injury is not serious.” Low-income households often reside in rural or underserved urban areas with limited healthcare facilities. This geographical barrier means that, even if they can afford healthcare, the physical distance to providers can be a significant obstacle.^17,18^ For many rural-dwelling Nigerians, the nearest qualified healthcare provider is hours away, complicating access to the necessary healthcare services.^52^ Furthermore, low health literacy, which is often associated with lower socioeconomic status, also contributes to unmet healthcare needs. Poor health literacy affects an individual’s ability to understand health information, navigate the healthcare system, and make informed decisions about their care, resulting in unmet healthcare needs.^17^ Given these challenges, improving healthcare access for low-income households – who tend to be less healthy and would benefit the most from healthcare – would lead to significant population health gains, along with substantial social and economic benefits.^18^

Consistent with previous studies, individuals with chronic illnesses had a higher prevalence of unmet healthcare needs.^31,40,44,45^ The cost of long-term and often expensive treatment for chronic illnesses is a significant barrier to accessing care for many Nigerians, especially those without insurance coverage. Even with insurance coverage, OOP copayments for such long-term illnesses leave many Nigerians with unmet healthcare needs.^40^ Additionally, healthcare facilities are absent in many communities, especially rural communities, making it difficult for residents with chronic illnesses to access necessary care. This difficulty is exacerbated by high transportation costs, poor road conditions, and a lack of transportation.^26,52^ To further complicate this, the successful treatment of chronic illnesses requires significant time commitments, including frequent medical appointments with long waiting times, repeated lab and pharmacy visits, and rehabilitation sessions. These demands are particularly challenging for Nigerians, who cannot take time off work.^51^ Furthermore, the stigma surrounding chronic illnesses such as TB, HIV, and mental health conditions remains strong in Nigeria, which may discourage individuals from seeking necessary care. Additionally, healthcare workforce shortages in Nigeria lead to inadequate staffing, hampering service delivery and increasing patient wait times.^53,54^ This issue is further complicated by poor quality of care due to outdated equipment and insufficient training, resulting in suboptimal outcomes and discouraging individuals from seeking medical attention.^26,52^ To effectively address these challenges, a comprehensive approach is required, which includes expanding insurance coverage, improving healthcare infrastructure, strengthening and updating the healthcare workforce, enhancing patient education, and reducing stigma.

This study highlights a significant link between household food insecurity and unmet healthcare needs, which is consistent with the findings of previous studies.^46,55-57^ Our analysis suggests that 73.9% of Nigerian households are food insecure, similar to the 73.4% reported in the 2021 National Health and Nutrition Survey.^58^ Approximately 8.4 million Nigerians (95% CI: 8.0-8.9 million) are experiencing both food insecurity and unmet healthcare needs. These Nigerians face a difficult choice between having enough to eat and seeking medical care. A hungry person is more concerned about their next meal than about early stage illnesses, which are often neglected until they become critical. Several studies have indicated that food insecurity leads to lower healthcare access for illnesses.^55,56^ Interestingly, we did not find a statistically significant relationship between severe food insecurity and unmet healthcare needs. This could be due to the significant effects of food insecurity on mental health across all genders and age groups, which seriously alters the perception of health and illness among those facing severe food insecurity.^59^ They might only seek healthcare when absolutely necessary and thus do not report unmet healthcare needs unless the situation is dire. Additionally, individuals experiencing severe food insecurity underreporting of unmet healthcare needs as they prioritize their immediate survival needs over seeking healthcare, even when it is critically needed.

## Strengths and Limitations

To the best of our knowledge, our study is the first to assess the prevalence of and inequalities in unmet healthcare needs in Nigeria. We utilized the most recent nationally representative publicly available data for our analysis. Additionally, we employed multilevel hierarchical regression analysis to effectively manage complex data groupings and nesting, providing clear insights into the influence of different levels on the study’s outcome. However, our study has some limitations that do not invalidate our findings. First, as our findings are based on cross-sectional data, establishing causality for the factors associated with unmet healthcare needs in the country remains challenging. Second, our study relied on data from 2018 to 2019, which may not fully reflect the current situation in the country, given the profound impact of the COVID-19 pandemic on health systems and global economies. The pandemic has likely exacerbated unmet healthcare needs due to increased strain on health services, disruptions in routine care, and economic hardships faced by the population. Therefore, our findings might underestimate the current extent of unmet healthcare needs in the country. Third, our dependent variable – unmet healthcare needs – is based on self-reported data, which may be subject to recall bias. Recall bias is a common issue in household surveys and can affect the accuracy of collected data. Moreover, self-reported unmet healthcare needs can be influenced by various unobservable factors, including cultural norms, health literacy levels, and personal expectations regarding health services. Fourth, the set of questions (health shock and healthcare-seeking behavior) used to assess our outcome variable (unmet need for healthcare) may not comprehensively cover all dimensions of unmet needs. Certain health conditions or services may be overlooked due to cultural norms and taboos. This is particularly significant considering the stigma associated with certain health conditions such as mental health and sexually transmitted infections (STIs). To the extent that this limitation – underreporting bias – affected our study, our prevalence estimate might represent the lower bounds of unmet healthcare needs in the country.

## Implications for Policy and Research

Our study provides valuable insights for policy discussions and health system reforms as Nigeria progresses toward UHC. First, it underscores the importance of regularly assessing unmet healthcare needs through household surveys in Nigeria and other countries that lack a social health insurance system. Perceived healthcare needs vary based on personal views and beliefs about what services are necessary and available. However, healthcare should always address individual needs. Since personal perceptions of health needs are the main motivators for seeking care, it is crucial to assess how well these patient-perceived needs are met.^60^ Evaluating how service capabilities and availability align with perceived healthcare needs can critically inform policymakers on the steps to strengthen the healthcare system. As a corollary, it is crucial to incorporate questions assessing unmet healthcare needs (beyond inquiries related to unmet need for contraception or family planning) in other large, publicly available standardized household surveys, such as the Demographic and Health Surveys and the Multiple Indicator Cluster Surveys. These surveys cover more confidential health topics, including reproductive health and STIs. By leveraging the comprehensive data gathered from these surveys, policymakers can gain detailed and nuanced insights into the underlying causes of unmet healthcare needs, which can inform more effective strategies to improve overall healthcare accessibility.

Second, the prevalence of unmet healthcare needs reflects an imbalance between the availability of healthcare services and the population’s demand for, access to, and satisfaction with these services.^13,61^ When the supply of healthcare services falls short, even with normal historical demand, the proportion of the population whose needs are met is less than ideal. Similarly, if the demand for healthcare services exceeds supply, some individuals will not meet their needs.^13^ The high prevalence of unmet healthcare needs identified in this study, along with Nigeria’s low UHC service coverage index, suggest a significant gap in the supply of healthcare services in the country.^5,6^ Given that Nigeria’s current health spending as a percentage of GDP is the second lowest in the world, our study underscores the urgent need for increased investment in healthcare infrastructure and resources, including funding of healthcare facilities, medical equipment, and trained healthcare personnel.^27^ Policies should aim to improve access to healthcare, particularly in underserved and rural areas, by expanding mobile clinics, telemedicine, and community health programs.^23,52,62^ Additionally, enhancing the quality of healthcare services through better provider training, adherence to clinical guidelines, and continuous monitoring and evaluation is crucial.^52^ On the demand side, our study also highlights the need for targeted interventions, such as subsidy/voucher schemes, cash transfer programs and community health programs, to ensure that vulnerable populations – such as the very poor, chronically ill, those living alone, and food insecure individuals – can effectively seek and access healthcare services.

Thirdly, the lack of significant associations between certain variables – such as education, region, and health insurance – and unmet healthcare needs in our adjusted models contrasts with existing literature.^21,22,30,31,40^ This discrepancy warrants further exploration. The lack of significant association with health insurance could be attributed to the very low insurance coverage in the country.^25^ Similarly, the lack of association with education might have been influenced by survey design or measurement issues.^21,40^ For instance, the categorization of education levels might have affected the results. Additionally, regional differences in unmet healthcare needs may be influenced by various contextual factors, including disparities in healthcare infrastructure, availability of services, cultural peculiarities, and socioeconomic conditions. These factors can vary significantly across regions and may not be fully captured by the variables included in our models. Future studies, preferably qualitative or mixed-method studies, should explore these contextual factors in greater detail to comprehensively understand their impact on unmet healthcare needs in similar settings. Future studies could also replicate this research by exploring additional variables, employing more advanced or alternative methodologies, or using larger samples in similar contexts. By pursuing these avenues, future research can build on the groundwork laid by this study and contribute to a more robust and nuanced understanding of the subject.

Finally, our study indicates that the determinants of unmet healthcare needs in Nigeria are multifactorial. Our multilevel models show that much of the variation in unmet healthcare needs stems from contextual factors, highlighting the significant impact of household- and community-level factors. Therefore, reducing disparities in accessing healthcare services, especially for vulnerable Nigerians, would require systemic reforms and multifaceted strategies that extend beyond the health sector, involving the input and coordination of other ministries, departments, and agencies. Strong investments in health, education, transportation infrastructure (especially in rural areas), basic public services, food distribution, and social services are required to improve the availability of healthcare services and boost Nigerians’ effective demand for them.^26^ Within the health sector, expanding health insurance coverage and reducing OOP healthcare payments at points of service is critical for improving healthcare access in Nigeria. The passage of the National Health Insurance Authority Act (NHIA) in 2022, which mandates health insurance for all Nigerians and legal residents and establishes a fund – the NHIA’s Vulnerable Group Fund – to support healthcare for vulnerable groups, is commendable.^27^ However, beyond passing the act, specific actionable steps to boost insurance coverage include reducing premiums (and paying the premiums for the poorest Nigerians), simplifying enrollment processes, and raising awareness about the benefits of insurance.^26^ Exploring alternative options, such as tax-funded noncontributory social health insurance, could be effective in improving overall access to healthcare services. Community-based health interventions improve access to healthcare services, especially in rural areas, through mobile clinics and telehealth, enhance health education, and build trust by involving local community members.^18,62^ These programs are also cost effective, address social determinants of health, strengthen local health systems, and improve healthcare access and health outcomes for all Nigerians.

## Conclusion

Access to healthcare is a crucial indicator of the effectiveness and efficiency of national and local health systems – reflecting how well these systems can provide necessary medical services to their populations – and unmet healthcare needs are increasingly being recognized as a key measure of access. This study revealed that a significant proportion of Nigerians have unmet healthcare needs, with poor and chronically ill individuals being disproportionately affected. Furthermore, living alone or in food-insecure households significantly predicted unmet healthcare needs in Nigeria. Policymakers should implement strategies to enhance healthcare accessibility, particularly for vulnerable Nigerians, to accelerate Nigeria’s progress toward universal health coverage.

## Supplemental Material

sj-docx-1-his-10.1177_11786329251330032 – Supplemental material for Understanding Unmet Healthcare Needs in Nigeria: Implications for Universal Health Coverage Supplemental material, sj-docx-1-his-10.1177_11786329251330032 for Understanding Unmet Healthcare Needs in Nigeria: Implications for Universal Health Coverage by Paul Eze, Chioma Lynda Aniebo, Stanley Ilechukwu and Lucky Osaheni Lawani in Health Services Insights

## Statements and Declarations

**Ethical Considerations:** The Pennsylvania State University’s research ethics committee waived the need for ethical approval and patient consent for the collection, analysis and publication of the retrospectively obtained and anonymized data for this non-interventional study.

**Consent to Participate:** Informed consent for the information published in this article was not obtained because our study data – NLSS 2018 to 2019 – has been anonymized and does not contain any personally identifiable information. Therefore, obtaining written informed consent from the study participants or the participants’ legal guardians/next of kin is both infeasible and unnecessary.

**Data Availability:** The datasets analyzed in this study are available on The World Bank Microdata Library, at https://doi.org/10.48529/8gbe-z155

**Supplemental Material:** Supplemental material for this article is available online.
