# Supplementary material for: Understanding Unmet Healthcare Needs in Nigeria: Implications for Universal Health Coverage
Source: Health Serv Insights. 2025 Mar 31;18:11786329251330032. doi: 10.1177/11786329251330032 (PMC11956516; doi:10.1177/11786329251330032)
Supplement: sj-docx-1-his-10.1177_11786329251330032 – Supplemental material for Understanding Unmet Healthcare Needs in Nigeria: Implications for Universal Health Coverage [file sj-docx-1-his-10.1177_11786329251330032.docx]

**Appendix/Supplementary files**

**Appendix 1:** Breakdown of reasons by education, wealth quintile, and residence (incorporating sample weights)

| **State** | **Cost-related, cannot afford** | **Illness or injury not severe** | **Health facilities are too far** | **Poor quality of healthcare** |
| --- | --- | --- | --- | --- |
| **Education** |  |  |  |  |
| - None | 16.4% | 80.7% | 6.0% | 1.2% |
| - Primary | 17.9% | 81.4% | 3.2% | 0.3% |
| - Secondary | 15.2% | 84.8% | 2.0% | 0.2% |
| - Post secondary | 14.1% | 80.8% | 0.1% | 5.0% |
| *p-value of chi-square test >>* | *0.762* | *0.604* | *<0.001* | *0.005* |
|  |  |  |  |  |
| **Wealth quintile** |  |  |  |  |
| - Poorest | 19.7% | 75.1% | 9.6% | 1.2% |
| - Poor | 14.2% | 84.1% | 3.5% | 1.1% |
| - Middle | 16.8% | 82.4% | 3.0% | 0.3% |
| - Rich | 13.4% | 86.5% | 2.0% | 0.4% |
| - Richest | 14.8% | 84.4% | 1.6% | 2.0%% |
| *p-value of chi-square test >>* | *0.231* | *0.003* | *<0.001* | *0.230* |
|  |  |  |  |  |
| **Residence** |  |  |  |  |
| - Rural | 17.2% | 79.5% | 5.9% | 1.4% |
| - Urban | 14.6% | 85.8% | 1.4% | 0.5% |
| *p-value of chi-square test >>* | *0.276* | *0.011* | *<0.001* | *0.056* |

**Appendix 2:** Breakdown of reasons for unmet healthcare needs by region (incorporating sample weights)

| **Region** | **Cost-related, cannot afford** | **Illness or injury not severe** | **Health facilities are too far** | **Poor quality of healthcare** |
| --- | --- | --- | --- | --- |
| - North Central | 17.0% | 82.1% | 2.9% | 0.1% |
| - North East | 10.9% | 84.4% | 9.6% | 0.7% |
| - North West | 13.8% | 81.3% | 3.9% | 2.7% |
| - South East | 14.8% | 84.2% | 0.0% | 1.9% |
| - South South | 22.2% | 78.3% | 4.1% | 0.6% |
| - South West | 17.9% | 81.1% | 2.4% | 0.7% |

**Appendix 3:** Breakdown of reasons for unmet healthcare needs by state (incorporating sample weights)

| **States** | **Region** | **Cost-related, cannot afford** | **Illness or injury not severe** | **Health facilities are too far** | **Poor quality of healthcare** |
| --- | --- | --- | --- | --- | --- |
| - Abia | South East | 16.2% | 76.9% | 0.0% | 7.0% |
| - Adamawa | North East | 48.4% | 50.8% | 0.0% | 6.4% |
| - Akwa Ibom | South South | 22.4% | 77.7% | 6.4% | 0.4% |
| - Anambra | South East | 8.3% | 91.7% | 0.0% | 1.0% |
| - Bauchi | North East | 11.9% | 86.1% | 2.0% | 0.3% |
| - Bayelsa | South South | 41.8% | 61.7% | 0.0% | 3.5% |
| - Benue | North Central | 25.8% | 74.5% | 2.1% | 0.0% |
| - Borno | North East | 6.4% | 89.5% | 5.6% | 0.3% |
| - Cross River | South South | 18.7% | 81.3% | 0.0% | 0.0% |
| - Delta | South South | 9.7% | 90.3% | 0.0% | 0.0% |
| - Ebonyi | South East | 28.6% | 73.9% | 0.0% | 0.0% |
| - Edo | South South | 9.4% | 93.5% | 0.0% | 0.0% |
| - Ekiti | South West | 18.0% | 77.0% | 5.0% | 0.0% |
| - Enugu | South East | 16.7% | 83.3% | 0.0% | 0.0% |
| - Gombe | North East | 11.1% | 87.6% | 4.6% | 0.4% |
| - Imo | South East | 11.5% | 88.5% | 0.0% | 0.0% |
| - Jigawa | North West | 7.0% | 80.7% | 4.2% | 9.9% |
| - Kaduna | North West | 41.5% | 58.5% | 0.0% | 0.0% |
| - Kano | North West | 14.9% | 80.9% | 4.4% | 2.2% |
| - Katsina | North West | 12.0% | 85.8% | 1.9% | 1.2% |
| - Kebbi | North West | 7.7% | 82.7% | 12.0% | 0.0% |
| - Kogi | North Central | 38.6% | 60.7% | 6.9% | 0.0% |
| - Kwara | North Central | 10.6% | 89.4% | 0.0% | 0.0% |
| - Lagos | South West | 31.5% | 68.5% | 0.0% | 0.0% |
| - Nasarawa | North Central | 5.8% | 94.0% | 0.0% | 1.2% |
| - Niger | North Central | 37.2% | 42.6% | 20.2% | 0.0% |
| - Ogun | South West | 25.2% | 68.3% | 12.4% | 3.7% |
| - Ondo | South West | 8.6% | 82.6% | 5.7% | 3.1% |
| - Osun | South West | 14.3% | 88.6% | 0.6% | 0.7% |
| - Oyo | South West | 13.9% | 85.4% | 1.7% | 0.0% |
| - Plateau | North Central | 10.2% | 87.8% | 3.3% | 0.0% |
| - Rivers | South South | 31.2% | 70.1% | 2.1% | 1.6% |
| - Sokoto | North West | 3.5% | 86.4% | 6.3% | 4.8% |
| - Taraba | North East | 7.0% | 81.9% | 11.4% | 1.1% |
| - Yobe | North East | 8.0% | 85.9% | 25.6% | 0.4% |
| - Zamfara | North West | 21.2% | 78.8% | 0.0% | 0.0% |
| - **FCT Abuja | North Central | 4.4% | 97.1% | 0.0% | 0.0% |

**Appendix 4:** Variance and model fit statistics of multilevel logistic models

|  | **Model 0**  **Null model** | **Model 1** | **Model 2** | **Model 3** | **Model 4**  **Full or Saturated** |
| --- | --- | --- | --- | --- | --- |
| **Variance of random effects** |  |  |  |  |  |
| - σ^2^ (v4) | 1.689 | 2.025 | 1.624 | 1.520 | 1.754 |
| - σ^2^ (v3) | 0.051 | 0.065 | 0.038 | 0.048 | 0.045 |
| - σ^2^ (v2) | 2.871 | 3.257 | 2.754 | 2.870 | 3.217 |
| - PCV – states | Reference | -19.9% | 3.8% | 10.0% | -3.9% |
| - PCV – communities | Reference | -25.8% | 26.1% | 6.8% | 11.7% |
| - PCV – households | Reference | -13.5% | 4.1% | 0.0% | -12.1% |
| - ICC – states | 0.214 | 0.235 | 0.211 | 0.200 | 0.211 |
| - ICC – community | 0.220 | 0.242 | 0.216 | 0.203 | 0.217 |
| - ICC – households | 0.584 | 0.619 | 0.573 | 0.574 | 0.604 |
| - MOR – states | 3.45 | 3.89 | 3.37 | 3.24 | 3.54 |
| - MOR – communities | 1.24 | 1.28 | 1.20 | 1.23 | 1.23 |
| - MOR – households | 5.03 | 5.59 | 4.87 | 5.03 | 5.53 |
| - LR test vs. logistic model | p < 0.001 | p < 0.001 | p < 0.001 | p < 0.001 | p < 0.001 |
|  |  |  |  |  |  |
| **Model fit statistics** |  |  |  |  |  |
| - Log likelihood | -19,223.80 | -17,823.39 | -17,880.27 | -19,217.73 | -16,589.21 |
| - Deviance | 38,447.60 | 35,646.78 | 35,760.54 | 38,435.46 | 33,178.42 |
| - LL ratio test |  | < 0.001 | < 0.001 | < 0.001 | < 0.001 |
| - AIC | 38,455.61 | 35,676.79 | 35,790.53 | 38,455.45 | 33,242.42 |
| - BIC | 38,494.26 | 35,821.75 | 35,934.38 | 38,552.10 | 33,549.30 |

PCV: proportional change of the variance, VPC: variance partition coefficient, ICC: intra class correlation, MOR: median odds ratio. σ^2^ (ν4), σ^2^ (ν3), and σ^2^ (ν2) are state, community, and household random intercept variances, respectively.

**Appendix 5**: Multilevel regression models for unmet healthcare needs in study participants

|  | **Model 0** | **Model 1**  **AOR (95% CI)** | **Model 2**  **AOR (95% CI)** | **Model 3**  **AOR (95% CI)** | **Model 4**  **AOR (95% CI)** |
| --- | --- | --- | --- | --- | --- |
| **Sex** |  |  |  |  |  |
| - Female |  | 1.01 (0.94, 1.08) |  |  | 1.03 (0.95, 1.10) |
| - Male |  | Reference |  |  | Reference |
| **Age** |  |  |  |  |  |
| - per 1-year increase |  | 1.00 (0.99, 1.01) |  |  | 1.00 (0.99, 1.00) ** |
| **Marital status** |  |  |  |  |  |
| - Single/Never married |  | Reference |  |  | Reference |
| - Married/Living as married |  | 0.88 (0.77, 1.01) |  |  | 0.93 (0.81, 1.07) |
| - Widowed/Divorced/Separated |  | 1.15 (0.92, 1.44) |  |  | 1.10 (0.87, 1.39) |
| **Religion** |  |  |  |  |  |
| - Christian |  | Reference |  |  | Reference |
| - Islam |  | 0.98 (0.83, 1.16) |  |  | 0.98 (0.83, 1.17) |
| - Others |  | 1.25 (0.78, 1.99) |  |  | 1.27 (0.79, 2.03) |
| **Education** |  |  |  |  |  |
| - None |  | Reference |  |  | Reference |
| - Primary |  | 0.95 (0.79, 1.13) |  |  | 0.89 (0.74, 1.08) |
| - Secondary |  | 1.06 (0.89, 1.26) |  |  | 0.99 (0.82, 1.19) |
| - Post-secondary |  | 1.14 (0.96, 1.35) |  |  | 1.05 (0.88, 1.26) |
| **Employment** |  |  |  |  |  |
| - No |  | Reference |  |  | Reference |
| - Yes |  | 1.10 (1.00, 1.19) ** |  |  | 1.05 (0.96, 1.15) |
| **Chronic illness** |  |  |  |  |  |
| - No |  | Reference |  |  | Reference |
| - Yes |  | 8.66 (7.95, 9.44) ** |  |  | 8.73 (7.99, 9.54) ** |
| **Household head** |  |  |  |  |  |
| - Female |  |  | 1.21 (1.05, 1.39) ** |  | 1.05 (0.89, 1.24) |
| - Male |  |  | Reference |  | Reference |
| **Household size** |  |  |  |  |  |
| - 1 person |  |  | 2.17 (1.72, 2.73) ** |  | 1.55 (1.20, 2.02) ** |
| - 2 to 5 persons |  |  | 1.17 (1.06, 1.30) ** |  | 1.10 (0.99, 1.23) |
| - >5 persons |  |  | Reference |  | Reference |
| **Wealth quintile** |  |  |  |  |  |
| - Poorest |  |  | 1.61 (1.35, 1.91) ** |  | 1.45 (1.19, 1.78) ** |
| - Poor |  |  | 1.23 (1.04, 1.47) ** |  | 1.12 (0.92, 1.37) |
| - Middle |  |  | 1.30 (1.10, 1.53) ** |  | 1.19 (0.99, 1.43) |
| - Rich |  |  | 1.19 (1.02, 1.39) ** |  | 1.17 (0.99, 1.38) |
| - Richest |  |  | Reference |  | Reference |
| **Health insurance** |  |  |  |  |  |
| - No |  |  | Reference |  | Reference |
| - Yes |  |  | 1.00 (0.74, 1.34) |  | 0.98 (0.71, 1.35) |
| **Household food security** |  |  |  |  |  |
| - Food secure |  |  | Reference |  | Reference |
| - Mildly food insecure |  |  | 1.21 (1.05, 1.39) ** |  | 1.17 (1.01, 1.36) ** |
| - Moderately food insecure |  |  | 1.33 (1.16, 1.53) ** |  | 1.30 (1.11, 1.51) ** |
| - Severely food insecure |  |  | 1.21 (1.03, 1.40) ** |  | 1.11 (0.94, 1.31) |
| **Residence** |  |  |  |  |  |
| - Rural |  |  |  | 1.18 (1.05, 1.32) | 1.09 (0.95, 1.25) |
| - Urban |  |  |  | Reference | Reference |
| **Geopolitical region** |  |  |  |  |  |
| - North Central |  |  |  | Reference | Reference |
| - North East |  |  |  | 1.89 (0.62, 5.77) | 1.60 (0.47, 5.53) |
| - North West |  |  |  | 2.05 (0.70, 6.02) | 2.26 (0.70, 7.36) |
| - South East |  |  |  | 0.79 (0.22, 2.88) | 0.51 (0.12, 2.10) |
| - South South |  |  |  | 2.92 (0.89, 9.58) | 2.27 (0.62, 8.38) |
| - South West |  |  |  | 1.59 (0.47, 5.36) | 1.54 (0.41, 5.73) |
| **Intercept coefficient (Std. error)** | -4.44961 (0.185295) | -5.19640 (0.224975) | -4.95288 (0.241723) | -5.03110 (0.418192) | -5.86873 (0.489791) |

AOR: Adjusted odds ratios, CI: Confidence interval

** p < 0.05
